# MRI-Based Structured Assessment of Treatment Response in Rectal Cancer After Total Neoadjuvant Therapy: Correlation With Surgical and Pathological Outcomes

**DOI:** 10.7759/cureus.113234

**Published:** 2026-07-23

**Authors:** Adyasha Kar, Manish Kumar Jha, Kashi Nath Sarkar, Sonali Priyadarshini, Manisha Sarkar, Shivani Sarkar

**Affiliations:** 1 Department of Radiodiagnosis, Raipur Institute of Medical Sciences, Raipur, IND; 2 Department of Radiodiagnosis, JIS School of Medical Science & Research, Howrah, IND; 3 Department of Community Medicine, Bankura Sammilani Medical College and Hospital, Bankura, IND; 4 Department of Oncopathology, Tata Medical Center, Kolkata, IND

**Keywords:** diffusion-weighted imaging (dwi), magnetic resonance imaging (mri), neoadjuvant therapy (nac), organ preservation, rectal cancer, structured reporting, surgical planning, total mesorectal excision, treatment response, watch and wait

## Abstract

Background

This study aimed to evaluate the utility of structured post-treatment magnetic resonance imaging (MRI) assessment for response evaluation in rectal cancer patients treated with total neoadjuvant therapy, with emphasis on surgical planning, histopathological correlation, and cautious multidisciplinary assessment of potential organ-preservation eligibility.

Methodology

This retrospective, observational cohort study included 100 patients with biopsy-proven rectal cancer who underwent baseline and post-treatment rectal MRI after total neoadjuvant therapy. Post-treatment imaging was reviewed using a structured response assessment template incorporating T2-weighted tumor-bed morphology, magnetic resonance tumor regression grade, diffusion restriction, mesorectal fascia status, extramural vascular invasion, nodal response, sphincter complex involvement, and pelvic sidewall disease. Imaging-based response categories were compared with operative and histopathological outcomes where available.

Results

Structured MRI assessment was performed in all 100 patients. Complete or near-complete imaging response was identified in 32 (32.0%) patients, incomplete response in 58 (58.0%) patients, and indeterminate response in 10 (10.0%) patients. Histopathological correlation was available in 84 surgically treated patients, of whom 21 (25.0%) patients had pathological complete response and 63 (75.0%) patients had residual viable tumor. When incomplete or indeterminate response was considered positive for residual disease, structured post-treatment MRI showed sensitivity of 88.9%, specificity of 81.0%, positive predictive value of 93.3%, negative predictive value of 70.8%, and accuracy of 86.9% for detecting residual viable tumor. Persistent intermediate T2 signal, focal diffusion restriction, residual extramural vascular invasion, and threatened mesorectal fascia were significantly associated with residual viable tumor. Exploratory predictive analysis showed strong associations of persistent intermediate T2 signal and focal diffusion restriction with residual viable tumor. Receiver operating characteristic analysis of the ordered structured MRI response category showed good discriminatory performance, with an area under the curve of 0.860. Sensitivity analyses showed that diagnostic performance varied according to the handling of indeterminate response, supporting cautious interpretation of equivocal post-treatment findings.

Conclusions

Structured post-treatment rectal MRI provides a practical framework for documenting response after total neoadjuvant therapy and communicating residual tumor morphology, diffusion restriction, mesorectal fascia status, extramural vascular invasion, nodal response, and sphincter or pelvic sidewall involvement to the multidisciplinary team. Although diagnostic performance was good in the surgically treated subgroup, findings should be interpreted cautiously because histopathological confirmation was unavailable in all patients, the watch-and-wait subgroup was small, follow-up was limited, and external validation was not performed. Structured MRI may support, but should not replace, integrated clinical, endoscopic, pathological, biochemical, and multidisciplinary assessment when considering organ-preserving management.

## Introduction

The management of locally advanced rectal cancer has changed substantially with the increasing use of total neoadjuvant therapy. Traditionally, magnetic resonance imaging (MRI) was used mainly for baseline local staging, assessment of mesorectal fascia involvement, extramural vascular invasion, nodal disease, sphincter complex involvement, and surgical planning. With total neoadjuvant therapy, MRI has acquired an additional role in post-treatment response assessment, because treatment decisions now extend beyond routine total mesorectal excision to include local excision and non-operative organ-preserving strategies in carefully selected patients [1-6].

Organ-preserving management has become increasingly relevant in patients who show clinical complete response or near-complete response after neoadjuvant treatment. The watch-and-wait approach requires careful patient selection and close surveillance, because local regrowth may occur and timely salvage treatment remains essential. Long-term observational data have shown that non-operative management can be feasible in selected complete responders when strict clinical, endoscopic, and imaging follow-up protocols are used [7,8].

Accurate assessment of treatment response after neoadjuvant therapy remains challenging. Post-treatment fibrosis, edema, mucinous degeneration, desmoplastic change, and inflammatory wall thickening may mimic residual tumor, while small foci of viable tumor may be difficult to identify within a fibrotic tumor bed. T2-weighted imaging provides anatomical detail and allows assessment of tumor regression morphology, mesorectal fascia clearance, sphincter complex involvement, and pelvic sidewall extension. MRI guidelines and previous rectal cancer imaging studies have emphasized the importance of standardized assessment of mesorectal fascia, extramural tumor spread, extramural vascular invasion, and tumor regression. Diffusion-weighted imaging (DWI) adds functional information and may improve detection of residual viable tumor, particularly when interpreted together with high-resolution T2-weighted images and apparent diffusion coefficient maps [9-16].

The emergence of watch-and-wait management has made radiologic response assessment more clinically relevant and more demanding. A complete or near-complete clinical response cannot be determined by MRI alone and requires integration with digital rectal examination, endoscopy, carcinoembryonic antigen level, and multidisciplinary review. However, MRI remains essential for identifying adverse residual features such as persistent intermediate T2 signal, focal diffusion restriction, residual extramural vascular invasion, suspicious mesorectal or lateral pelvic nodes, threatened mesorectal fascia, and persistent sphincter or levator involvement. Recent literature on rectal MRI response assessment has highlighted the need for careful interpretation after neoadjuvant therapy because post-treatment pitfalls may affect selection for surgery, local excision, or organ-preserving surveillance [17-20]. Studies evaluating DWI, combined T2-weighted and diffusion-weighted imaging interpretation, and standardized assessment of complete or near-complete response have shown that MRI can improve response assessment but cannot reliably confirm complete response in isolation. Reader variability, overlap between fibrosis and residual viable tumor, equivocal diffusion signal, and absence of uniform long-term outcome validation remain important limitations. Therefore, post-treatment MRI is best interpreted as one component of integrated multidisciplinary response assessment rather than as an independent determinant of watch-and-wait eligibility.

Despite the central role of MRI, post-treatment rectal cancer reports may vary considerably in terminology, completeness, and clinical usefulness. A structured approach can reduce reporting variability by ensuring that key response parameters are evaluated consistently and communicated clearly to the multidisciplinary team. Structured reporting is particularly important in the era of total neoadjuvant therapy, where the radiologist is expected not only to describe residual disease but also to help guide individualized management.

Although several established MRI-based frameworks, including European Society of Gastrointestinal and Abdominal Radiology (ESGAR) recommendations, Magnetic Resonance Imaging and Rectal Cancer European Equivalence (MERCURY)-based assessment, MRI-based tumor regression grade (mrTRG) grading, and DWI response assessment, provide important guidance for rectal cancer staging and restaging, post-treatment reporting after total neoadjuvant therapy remains variable in routine clinical practice. Existing frameworks often emphasize selected components such as tumor regression, mesorectal fascia status, diffusion restriction, or nodal response, but a practical consolidated template that systematically documents all key post-treatment findings relevant to surgery and organ-preservation decisions is still needed. This study addresses this gap by evaluating a structured institutional MRI response assessment template that integrates T2-weighted tumor-bed morphology, DWI, mesorectal fascia clearance, extramural vascular invasion response, nodal response, sphincter and levator involvement, pelvic sidewall disease, and management-relevant response categories in a single reporting framework.

This study aimed to evaluate the utility of a structured post-treatment MRI assessment template for rectal cancer patients treated with total neoadjuvant therapy. The objectives were to assess MRI-based treatment response using T2-weighted morphology, DWI, mesorectal fascia status, extramural vascular invasion, nodal response, and sphincter or pelvic sidewall involvement, and to correlate structured MRI response categories with surgical and histopathological outcomes.

## Materials and methods

Study design and ethical approval

This retrospective observational cohort study was conducted in the Department of Radiodiagnosis, Raipur Institute of Medical Sciences, Raipur, Chhattisgarh, India. The study protocol was reviewed and approved by the Institutional Ethics Committee of Raipur Institute of Medical Sciences, Raipur (approval number: IEC/RIMS/2024/03, dated 05/01/2024). The approved study title was “MRI-Based Structured Assessment of Treatment Response in Rectal Cancer After Total Neoadjuvant Therapy: Correlation With Surgical and Pathological Outcomes.” Patient confidentiality was maintained throughout the study, and all clinical, imaging, surgical, and histopathological data were anonymized before analysis.

Patient selection

The institutional radiology and oncology databases were reviewed to identify all consecutive patients with biopsy-proven rectal adenocarcinoma who underwent baseline and post-treatment rectal MRI after total neoadjuvant therapy between January 2024 and March 2025. A total of 132 patients were initially assessed for eligibility. Of these, 32 patients were excluded because of predefined exclusion criteria, including unavailable or technically inadequate baseline or post-treatment MRI in eight patients, tumor not located in the rectum in four patients, unavailable histopathological confirmation in six patients, prior pelvic surgery or recurrent rectal cancer before treatment in five patients, and incomplete essential clinical, operative, or histopathological records in nine patients. After these exclusions, 100 consecutive eligible patients were included in the final study cohort.

Patients were eligible if they had histologically confirmed rectal adenocarcinoma, baseline pelvic MRI performed before initiation of neoadjuvant therapy, completion of total neoadjuvant therapy, and post-treatment rectal MRI performed before definitive management. Definitive management included total mesorectal excision, local excision, or multidisciplinary selection for non-operative watch-and-wait surveillance.

**Figure 1:**
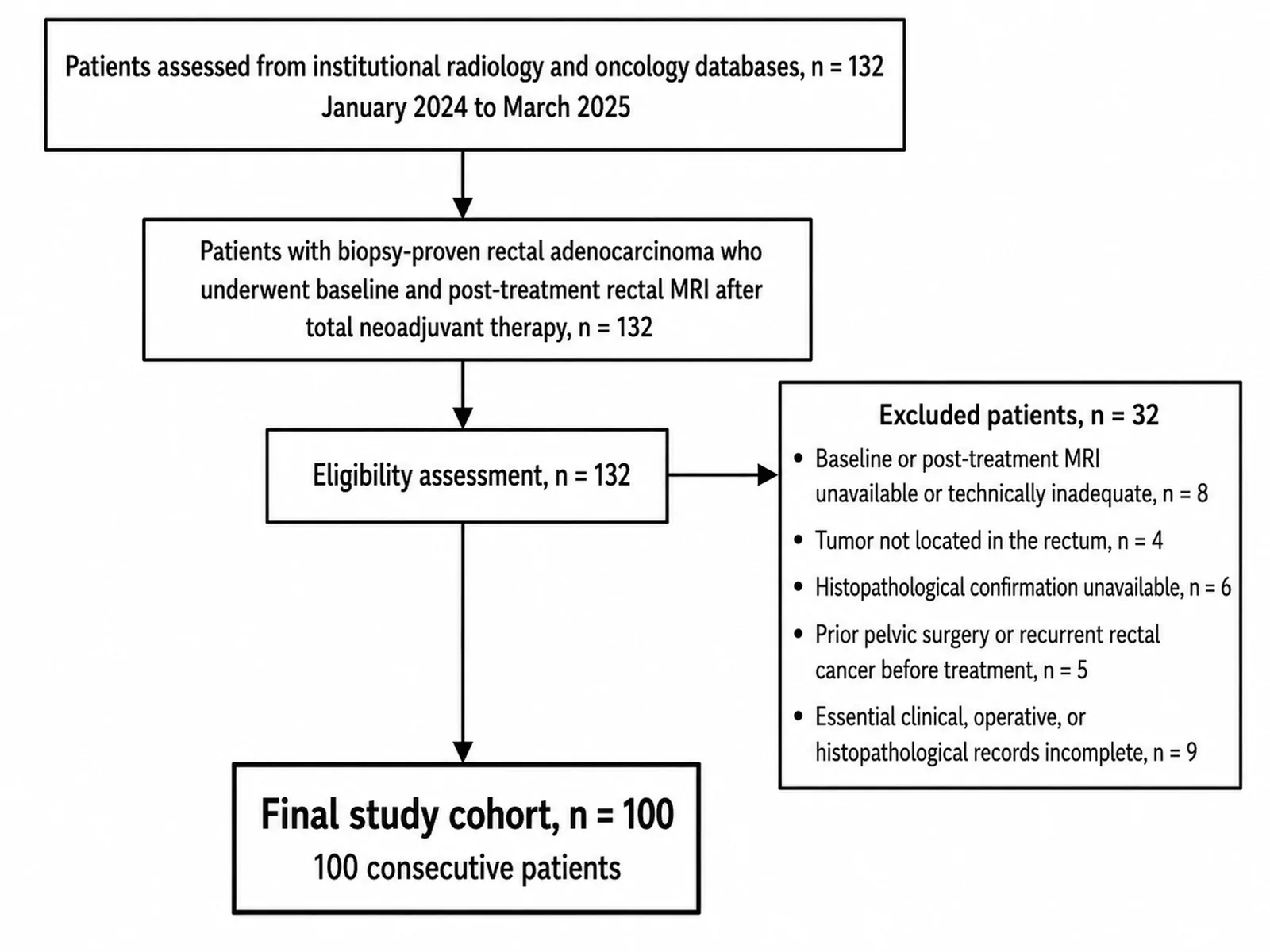
Flow diagram of patient selection. Flow diagram showing identification and selection of patients with biopsy-proven rectal adenocarcinoma who underwent baseline and post-treatment rectal magnetic resonance imaging after total neoadjuvant therapy between January 2024 and March 2025. A total of 132 patients were assessed from institutional radiology and oncology databases, of whom 32 patients were excluded according to predefined exclusion criteria, resulting in a final study cohort of 100 consecutive patients. Data are represented as n. No statistical comparison was performed for this figure. MRI = magnetic resonance imaging

Patient age, sex, clinical presentation, tumor location, baseline clinical stage, neoadjuvant treatment regimen, interval between completion of neoadjuvant therapy and post-treatment MRI, type of definitive management, operative findings, and histopathological outcomes were recorded from electronic medical records. Additional clinical variables recorded for exploratory interpretation included baseline T stage, baseline nodal status, tumor distance from the anal verge, tumor location, total neoadjuvant therapy regimen, pretreatment carcinoembryonic antigen level where available, treatment-to-MRI interval, and final management pathway. These variables were considered clinically relevant because they may influence treatment response and residual viable tumor after total neoadjuvant therapy. The patient selection process is summarized in Figure 1.

Magnetic resonance imaging protocol

All MRI examinations were performed on a 3-T MRI scanner using a phased-array pelvic coil according to the institutional rectal MRI protocol. High-resolution T2-weighted images were obtained in sagittal, oblique axial, and oblique coronal planes. Oblique axial images were planned perpendicular to the long axis of the tumor and rectal lumen, and oblique coronal images were planned parallel to the tumor axis, particularly for low rectal tumors and assessment of sphincter complex involvement. The high-resolution T2-weighted sequences were acquired with thin sections, small field of view, and high matrix resolution to allow assessment of tumor-bed morphology, mesorectal fascia, extramural disease, sphincter complex, levator involvement, and pelvic sidewall extension.

DWI was obtained in the axial plane using multiple b values, including b 0, b 500, and b 1,000 s/mm², with automatically generated apparent diffusion coefficient maps. Diffusion restriction was assessed qualitatively by identifying focal high signal on high b-value diffusion-weighted images with corresponding low signal on apparent diffusion coefficient maps at the treated tumor bed or extramural component. Apparent diffusion coefficient maps were used for lesion confirmation and correlation with T2-weighted morphology rather than as an isolated quantitative measurement. Axial pelvic T1-weighted and T2-weighted sequences were also obtained for assessment of lymph nodes, pelvic sidewall disease, bone marrow, pelvic organs, and associated pelvic findings. Intravenous contrast-enhanced imaging was performed when clinically indicated according to institutional protocol.

Baseline MRI was used to document pretreatment tumor morphology, tumor height from the anal verge, craniocaudal tumor length, circumferential location, T stage, mesorectal fascia involvement, extramural vascular invasion, mesorectal nodal disease, lateral pelvic nodal disease, sphincter complex involvement, and pelvic sidewall involvement. Post-treatment MRI was used for structured response assessment and was interpreted in direct comparison with the baseline study.

The minimum imaging parameters used for post-treatment response assessment are summarized in Table 1.

**Table 1:** Post-treatment rectal magnetic resonance imaging parameters used for structured response assessment. This table summarizes the minimum post-treatment rectal magnetic resonance imaging parameters evaluated in the structured response assessment template after total neoadjuvant therapy. Data are represented as descriptive imaging parameters and clinical relevance categories; no statistical comparison was performed for this table. A p-value less than 0.05 was considered statistically significant where comparative testing was performed. Note: Findings should be interpreted by direct comparison with baseline MRI and correlated with clinical examination, endoscopy, operative findings, and histopathology when available. ADC = apparent diffusion coefficient; DWI = diffusion-weighted imaging; EMVI = extramural vascular invasion; MRI = magnetic resonance imaging

MRI parameter	Sequence used	Finding assessed	Clinical relevance
Treated primary tumor bed	High-resolution T2-weighted imaging	Low T2 signal fibrosis, persistent intermediate tumor signal, nodular wall thickening	Differentiates favorable response from suspected residual tumor
Diffusion restriction	High b-value DWI and ADC map	Focal high signal on DWI with corresponding low ADC signal	Suggests residual viable tumor when correlated with T2 morphology
Mesorectal fascia	Oblique axial and coronal T2-weighted imaging	Distance of residual tumor or suspicious extramural disease from mesorectal fascia	Predicts circumferential resection margin risk
Extramural vascular invasion	High-resolution T2-weighted imaging with DWI correlation	Persistent irregular or expanded extramural vascular tumor signal	Indicates adverse residual risk feature
Mesorectal lymph nodes	Axial and oblique T2-weighted imaging with DWI	Size, morphology, border irregularity, heterogeneity, interval response	Assesses residual nodal disease
Lateral pelvic lymph nodes	Axial pelvic T2-weighted imaging and DWI	Persistent suspicious internal iliac, obturator, or external iliac nodes	Influences pelvic sidewall management
Sphincter complex and levator	Coronal and axial T2-weighted imaging	Residual involvement of internal sphincter, intersphincteric plane, external sphincter, or levator	Guides surgical approach and organ preservation suitability
Pelvic sidewall disease	Axial and coronal T2-weighted imaging	Residual extension to pelvic sidewall structures	Identifies locally advanced residual disease

Structured magnetic resonance imaging assessment

Post-treatment MRI examinations were reviewed using a structured response assessment template designed for rectal cancer after total neoadjuvant therapy. The template included residual tumor morphology on T2-weighted imaging, magnetic resonance tumor regression grade, diffusion restriction, mesorectal fascia status, extramural vascular invasion, mesorectal nodal response, lateral pelvic nodal status, sphincter complex involvement, levator involvement, pelvic sidewall disease, and other pelvic findings relevant to surgical planning.

On T2-weighted imaging, complete or near-complete response was considered when the previous tumor bed showed predominant low-signal fibrosis, marked reduction or disappearance of intermediate tumor signal, restoration of rectal wall architecture, and no definite residual extramural tumor. Incomplete response was suspected when persistent intermediate T2 signal, nodular residual wall thickening, persistent extramural soft tissue, irregular tumor-like signal, or progressive lesion bulk was present.

DWI was interpreted together with apparent diffusion coefficient maps. Definite focal high signal on high b-value DWI with corresponding low signal on apparent diffusion coefficient maps at the treated tumor site was considered suspicious for residual viable tumor. Linear, diffuse, or ill-defined diffusion signal without a corresponding focal low apparent diffusion coefficient correlate was not considered definite residual disease.

Magnetic resonance tumor regression grade was assigned according to the relative proportion of fibrosis and residual tumor signal within the treated tumor bed. Response patterns showing predominant fibrosis were considered favorable, whereas response patterns showing obvious residual intermediate tumor signal were considered unfavorable. The structured response assessment framework is summarized in Table 2.

**Table 2:** Structured magnetic resonance imaging response categories after total neoadjuvant therapy. This table summarizes the structured post-treatment rectal magnetic resonance imaging response categories used for assessment after total neoadjuvant therapy. Data are represented as descriptive categorical response criteria and management implications; no statistical comparison was performed for this table. A p-value less than 0.05 was considered statistically significant where comparative testing was performed. ADC = apparent diffusion coefficient; DWI = diffusion-weighted imaging; EMVI = extramural vascular invasion; MRI = magnetic resonance imaging; TNT = total neoadjuvant therapy

Response category	T2-weighted finding	Diffusion-weighted imaging finding	Mesorectal fascia, EMVI, and nodal finding	Management implication
Complete or near-complete response	Predominant low T2 signal fibrosis at the treated tumor bed with absent or only minimal residual intermediate signal; restoration or near-restoration of rectal wall architecture; no definite nodular residual extramural tumor	No definite focal restricted diffusion at the treated tumor site or extramural component; no focal high signal on high b-value images with corresponding low ADC signal	No suspicious residual EMVI; no threatened mesorectal fascia by residual tumor; no suspicious residual mesorectal or lateral pelvic nodes	May support consideration of organ preservation or watch-and-wait after correlation with digital rectal examination, endoscopy, tumor marker status, and multidisciplinary consensus
Incomplete response	Persistent intermediate T2 tumor-like signal, nodular residual wall thickening, residual extramural soft tissue, progressive lesion bulk, or persistent involvement of sphincter, levator, or pelvic sidewall structures	Definite focal restricted diffusion at the tumor bed or extramural component, especially when corresponding to persistent intermediate T2 signal	Persistent or recurrent EMVI, suspicious residual mesorectal or lateral pelvic nodes, or residual tumor threatening the mesorectal fascia may be present	Supports total mesorectal excision, local surgical planning, or further oncologic discussion depending on tumor location, residual risk features, and patient factors
Indeterminate response	Mixed fibrosis and equivocal intermediate signal; post-treatment edema, mucinous change, inflammation, or artifact limiting confident separation of fibrosis from residual tumor	Equivocal, non-specific, diffuse, or linear diffusion signal without a definite focal low ADC correlate	Uncertain mesorectal fascia, EMVI, or nodal status; findings do not clearly meet criteria for favorable or incomplete response	Requires multidisciplinary correlation, short-interval repeat MRI, endoscopic reassessment, biopsy when appropriate, or close surveillance before definitive organ-preservation decisions

The complete structured post-treatment rectal MRI reporting template used in this study is provided in Appendix 1 to facilitate clinical application and reproducibility.

Assessment of mesorectal fascia, extramural vascular invasion, and nodal response

The relationship of residual tumor or post-treatment fibrosis to the mesorectal fascia was recorded on post-treatment MRI. The mesorectal fascia was considered threatened when residual tumor signal or suspicious extramural disease was located within 1 mm of the mesorectal fascia. Fibrotic tissue alone was separately documented to reduce overstaging when no definite viable tumor signal was identified.

Extramural vascular invasion was assessed by evaluating treated or residual tumor signal within extramural vessels. Persistent expanded, nodular, or irregular extramural vascular signal showing intermediate T2 signal and/or diffusion restriction was considered suspicious for residual extramural vascular invasion. Thin low-signal fibrotic vascular strands without suspicious diffusion restriction were recorded as treated extramural vascular invasion.

Mesorectal and lateral pelvic lymph nodes were assessed for short-axis size, border irregularity, signal heterogeneity, morphology, diffusion restriction, and interval response compared with baseline imaging. Nodes that decreased in size and became homogeneous or fibrotic were considered responding nodes. Nodes with persistent suspicious morphology, irregular border, heterogeneous signal, or restricted diffusion were considered suspicious residual nodes.

Response categorization

Each patient was assigned to one of three imaging response categories: complete or near-complete response, incomplete response, or indeterminate response. These response categories were not intended to replace established ESGAR, MERCURY, or mrTRG-based rectal MRI assessment systems. They were adapted from established principles of post-treatment rectal MRI interpretation, including T2-weighted tumor regression morphology, mrTRG-related fibrosis-versus-residual-tumor assessment, DWI correlation, mesorectal fascia evaluation, extramural vascular invasion assessment, and nodal response criteria. These established components were consolidated into a practical institutional structured response classification to support multidisciplinary communication and management-oriented reporting after total neoadjuvant therapy.

**Figure 2:**
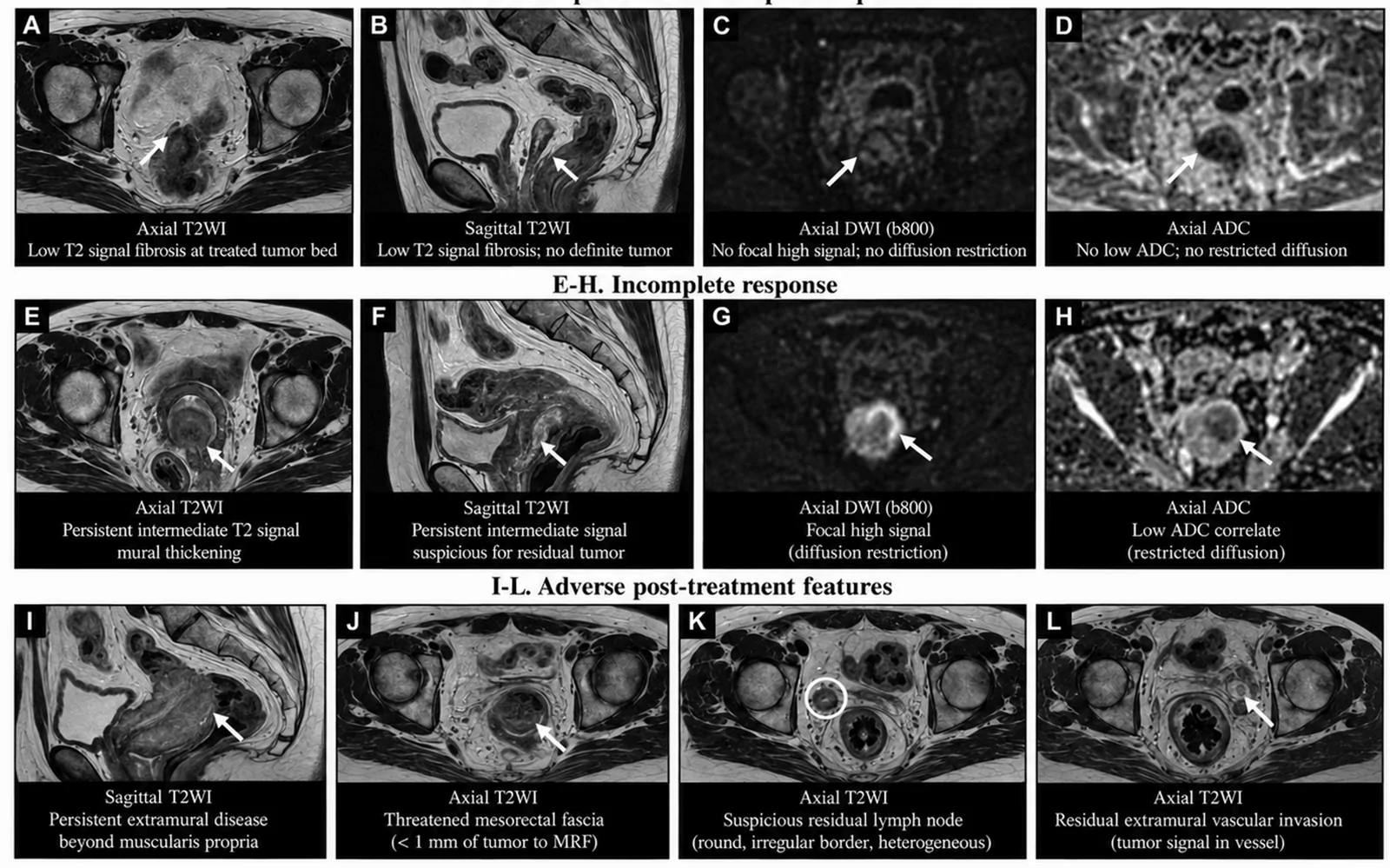
Representative post-treatment magnetic resonance imaging response patterns in rectal cancer. (A-D) Complete or near-complete response showing low T2 signal fibrosis at the treated tumor bed without definite focal diffusion restriction. Panel A shows axial T2-weighted imaging with low-signal fibrosis at the treated tumor bed. Panel B shows sagittal T2-weighted imaging with low T2 signal fibrosis and no definite residual tumor. Panel C shows axial diffusion-weighted imaging with no focal high signal. Panel D shows the corresponding axial apparent diffusion coefficient map without low signal, indicating absence of restricted diffusion. (E-H) Incomplete response showing persistent intermediate T2 signal and corresponding focal diffusion restriction suspicious for residual viable tumor. Panel E shows axial T2-weighted imaging with persistent intermediate T2 signal mural thickening. Panel F shows sagittal T2-weighted imaging with persistent intermediate signal suspicious for residual tumor. Panel G shows axial diffusion-weighted imaging with focal high signal at the treated tumor bed. Panel H shows the corresponding axial apparent diffusion coefficient map with low signal, indicating restricted diffusion. (I-L) Adverse post-treatment features. Panel I shows sagittal T2-weighted imaging with persistent extramural disease beyond the muscularis propria. Panel J shows axial T2-weighted imaging with threatened mesorectal fascia. Panel K shows axial T2-weighted imaging with a suspicious residual lymph node. Panel L shows axial T2-weighted imaging with residual extramural vascular invasion. ADC = apparent diffusion coefficient; DWI = diffusion-weighted imaging; EMVI = extramural vascular invasion; MRI = magnetic resonance imaging; T2WI = T2-weighted imaging Figure panels are representative imaging examples. No statistical comparison was performed for this figure.

Complete or near-complete response required predominant fibrosis at the primary tumor bed, absence of definite focal diffusion restriction, no suspicious residual extramural vascular invasion, no threatened mesorectal fascia by residual tumor, and no suspicious residual nodal disease. Incomplete response was assigned when persistent tumor-like intermediate T2 signal, focal diffusion restriction, residual extramural vascular invasion, suspicious residual lymph node, threatened mesorectal fascia, or persistent sphincter, levator, or pelvic sidewall involvement was present. Indeterminate response was assigned when imaging findings were equivocal because of post-treatment edema, mucinous change, motion artifact, or difficulty distinguishing fibrosis from residual tumor. Representative imaging response patterns are shown in Figure 2.

Surgical and histopathological correlation

For patients who underwent surgery, operative notes and histopathology reports were reviewed. Histopathological variables included pathological T stage, pathological N stage, pathological tumor regression grade, pathological complete response, residual viable tumor, lymphovascular invasion, perineural invasion, circumferential resection margin status, distal margin status, and quality of total mesorectal excision when reported. Pathological complete response was defined as absence of viable tumor cells in the resected primary tumor bed and regional lymph nodes.

For patients managed with watch-and-wait surveillance, clinical examination, endoscopic assessment, and follow-up MRI findings were recorded. These patients were not used as a pathological reference standard unless subsequent local regrowth or surgery provided histopathological confirmation.

Diagnostic performance analysis was restricted to the 84 surgically treated patients with a histopathological reference standard. Patients managed with watch-and-wait surveillance were not included in sensitivity, specificity, positive predictive value, negative predictive value, or accuracy calculations unless histopathological confirmation became available. This approach avoided treating clinical follow-up as equivalent to histopathology; however, it may introduce verification and selection bias because patients selected for non-operative management were more likely to have favorable clinical, endoscopic, and imaging responses. Therefore, diagnostic accuracy results were interpreted as applicable to the surgically treated subgroup rather than the entire 100-patient cohort.

Observer review

MRI examinations were reviewed independently by two radiologists with 12 years and 6 years of experience in abdominal and oncologic imaging. Before formal review, both readers underwent a calibration session using the structured response template and representative post-treatment rectal MRI examinations not included in the final analysis. Standardized interpretation criteria were reviewed for low T2 signal fibrosis, persistent intermediate T2 tumor-like signal, focal restricted diffusion, treated versus residual extramural vascular invasion, threatened mesorectal fascia, suspicious mesorectal or lateral pelvic lymph nodes, sphincter or levator involvement, and pelvic sidewall disease. The readers were aware that all patients had rectal cancer treated with total neoadjuvant therapy but were blinded to final histopathological outcomes during independent imaging review. Each reader independently assessed the overall imaging response category, magnetic resonance tumor regression pattern, diffusion restriction, mesorectal fascia status, extramural vascular invasion, and nodal response. Interobserver agreement was calculated using the independent reader assessments before any consensus discussion. After interobserver agreement analysis was completed, discrepancies were resolved by consensus to generate the final imaging assessment used for clinicopathological correlation. Because the final clinicopathological correlation was based on consensus interpretation, consensus review may have improved apparent diagnostic performance and was considered a potential source of observer-related bias.

Statistical analysis

Statistical analysis was performed using SPSS Statistics version 26.0 (IBM Corp., Armonk, NY, USA). Continuous variables were summarized as mean ± standard deviation when normally distributed and as median with interquartile range when not normally distributed. Categorical variables were summarized as number and percentage, expressed as n (%). Because the study was retrospective and observational, statistical testing was selected according to the data type and the objective of comparison.

Categorical imaging findings were compared with histopathological outcome using the Pearson chi-square test. Fisher’s exact test was considered when expected cell counts were small. For comparisons involving continuous variables between two independent groups, the independent-samples t-test was planned for normally distributed variables, and the Mann-Whitney U test was planned for non-normally distributed variables. Diagnostic performance of structured post-treatment MRI for identifying residual viable tumor was calculated using histopathology as the reference standard in the surgically treated subgroup. Sensitivity, specificity, positive predictive value, negative predictive value, and accuracy were calculated with 95% confidence intervals.

Interobserver agreement was assessed using Cohen kappa statistics for categorical variables and weighted kappa statistics for ordinal variables, with 95% confidence intervals reported where calculable. Kappa agreement was interpreted as poor, fair, moderate, substantial, or almost perfect. A two-sided p-value less than 0.05 was considered statistically significant.

Exploratory predictive analysis was performed in the surgically treated subgroup using histopathological residual viable tumor as the outcome. Univariable odds ratios with 95% confidence intervals were calculated for imaging predictors significantly associated with residual viable tumor. Variables considered for exploratory modeling included persistent intermediate T2 signal, focal diffusion restriction, residual extramural vascular invasion, threatened mesorectal fascia, suspicious residual lymph nodes, baseline T stage, baseline nodal status, tumor location, total neoadjuvant therapy regimen, pretreatment carcinoembryonic antigen level where available, and treatment-to-MRI interval. Because the number of pathological complete response events was limited and several imaging variables were overlapping components of the structured MRI response category, formal multivariable logistic regression was not used to create a definitive clinical prediction model. Receiver operating characteristic analysis was performed using the structured MRI response category as an ordinal predictor of residual viable tumor, and the area under the curve was calculated. Internal validation of the ordinal response-category model was performed using bootstrap resampling. Sensitivity analyses were performed by treating indeterminate MRI response as positive for residual disease, excluding indeterminate cases, and treating indeterminate MRI response as negative for residual disease.

## Results

Patient cohort

A total of 100 consecutive patients with biopsy-proven rectal adenocarcinoma who underwent baseline and post-treatment rectal MRI after total neoadjuvant therapy were included in the final analysis. The patient selection process is shown in Figure 1. The mean patient age was 56.8 ± 11.4 years. The cohort included 64 (64.0%) men and 36 (36.0%) women. Tumors were located in the lower rectum in 38 (38.0%) patients, mid rectum in 44 (44.0%) patients, and upper rectum in 18 (18.0%) patients. On baseline MRI, 8 (8.0%) patients had cT2 disease, 72 (72.0%) patients had cT3 disease, and 20 (20.0%) patients had cT4 disease. Baseline nodal positivity was present in 78 (78.0%) patients, while 22 (22.0%) patients were node negative. The baseline demographic and imaging characteristics are summarized in Table 3.

**Table 3:** Baseline clinical and magnetic resonance imaging characteristics of the study cohort. This table summarizes the baseline demographic, clinical, and magnetic resonance imaging characteristics of the 100 patients included in the study. Data are represented as n (%) for categorical variables and mean ± standard deviation for age. A p-value less than 0.05 was considered statistically significant where comparative testing was performed. MRI = magnetic resonance imaging; TNT = total neoadjuvant therapy

Variable	Category/Finding	Value
Total patients	Final study cohort	100
Age	Mean ± standard deviation, years	56.8 ± 11.4
Sex	Male	64 (64.0%)
	Female	36 (36.0%)
Tumor location	Lower rectum	38 (38.0%)
	Mid rectum	44 (44.0%)
	Upper rectum	18 (18.0%)
Baseline clinical T stage	cT2	8 (8.0%)
	cT3	72 (72.0%)
	cT4	20 (20.0%)
Baseline nodal status	Node-positive disease	78 (78.0%)
	Node-negative disease	22 (22.0%)

Post-treatment magnetic resonance imaging response

Structured post-treatment MRI assessment was successfully performed in all 100 patients using the predefined response parameters described in Table 1 and the response categorization framework shown in Table 2. Complete or near-complete imaging response was identified in 32 (32.0%) patients, incomplete response in 58 (58.0%) patients, and indeterminate response in 10 (10.0%) patients.

Among the 32 (32.0%) patients with complete or near-complete imaging response, the treated tumor bed showed predominant low T2 signal fibrosis, marked regression of tumor bulk, absence of definite focal diffusion restriction, and no suspicious residual extramural vascular invasion or threatened mesorectal fascia. Representative complete or near-complete response patterns are shown in Figures 2A-2D.

Among the 58 (58.0%) patients with incomplete imaging response, persistent intermediate T2 signal at the treated tumor bed was observed in 50 (86.2%) patients, focal diffusion restriction in 47 (81.0%) patients, residual extramural vascular invasion in 22 (37.9%) patients, suspicious residual mesorectal or lateral pelvic lymph nodes in 31 (53.4%) patients, and threatened mesorectal fascia in 18 (31.0%) patients. Representative incomplete response and adverse post-treatment imaging features are shown in Figures 2E-2L.

Definitive management and histopathological outcomes

Of the 100 patients, 78 (78.0%) patients underwent total mesorectal excision, 6 (6.0%) patients underwent local excision, and 16 (16.0%) patients were selected for non-operative watch-and-wait surveillance after multidisciplinary assessment. Histopathological correlation was available in 84 surgically treated patients (84.0%). Among these 84 patients, pathological complete response was observed in 21 (25.0%) patients, while residual viable tumor was identified in 63 (75.0%) patients. Pathological nodal positivity was present in 28 (33.3%) patients. Circumferential resection margin involvement was identified in 7 (8.3%) patients, all of whom had incomplete or indeterminate response on post-treatment MRI.

In the surgical subgroup, complete or near-complete imaging response was observed in 24 (28.6%) patients. Of these, 17 (70.8%) patients had pathological complete response and 7 (29.2%) patients had residual microscopic or small-volume viable tumor. Incomplete imaging response was observed in 52 (61.9%) surgically treated patients, of whom 49 (94.2%) patients had residual viable tumor and 3 (5.8%) patients had pathological complete response. Indeterminate imaging response was observed in 8 (9.5%) surgically treated patients, of whom 7 (87.5%) patients had residual viable tumor, and 1 (12.5%) patient had pathological complete response.

Correlation of imaging findings with residual disease

Persistent intermediate T2 signal, focal diffusion restriction, residual extramural vascular invasion, suspicious residual lymph nodes, and threatened mesorectal fascia were more frequently observed in patients with residual viable tumor than in those with pathological complete response. Focal diffusion restriction was present in 47 of 63 patients (74.6%) with residual viable tumor and in 3 of 21 patients (14.3%) with pathological complete response. This association was statistically significant on Pearson chi-square testing (χ² = 23.784, p < 0.001). Persistent intermediate T2 signal was present in 52 of 63 patients (82.5%) with residual viable tumor and in 4 of 21 patients (19.0%) with pathological complete response, also showing a statistically significant association with residual viable tumor on Pearson chi-square testing (χ² = 28.571, p < 0.001).

Residual extramural vascular invasion was present in 22 of 63 patients (34.9%) with residual viable tumor and in 0 of 21 patients (0.0%) with pathological complete response. This association was statistically significant on Fisher’s exact testing (p = 0.001). Threatened mesorectal fascia was present in 18 of 63 patients (28.6%) with residual viable tumor and in 0 of 21 patients (0.0%) with pathological complete response, showing a statistically significant association on Fisher’s exact testing (p = 0.004). These findings indicate that persistent intermediate T2 signal, focal diffusion restriction, residual extramural vascular invasion, and threatened mesorectal fascia were significantly associated with residual viable tumor on histopathology.

When incomplete or indeterminate response was considered positive for significant residual disease, structured MRI showed a sensitivity of 88.9%, specificity of 81.0%, positive predictive value of 93.3%, negative predictive value of 70.8%, and overall accuracy of 86.9% for detecting residual viable tumor in the surgical cohort. The association between structured MRI response category and histopathological residual viable tumor was statistically significant on Pearson chi-square testing (χ² = 37.644, p < 0.001). The diagnostic performance of structured post-treatment MRI is summarized in Table 4, and the association between structured MRI response category and histopathological outcome is shown in Table 5.

**Table 4:** Diagnostic performance of structured post-treatment magnetic resonance imaging for detection of residual viable tumor. This table summarizes the diagnostic performance of structured post-treatment magnetic resonance imaging for identifying residual viable tumor using histopathology as the reference standard in surgically treated patients. Data are represented as numerator/denominator, percentage, and 95% confidence interval. Statistical comparison is not applicable to this diagnostic performance table. A p-value less than 0.05 was considered statistically significant where comparative testing was performed. CI = confidence interval; MRI = magnetic resonance imaging

Diagnostic parameter	Numerator/Denominator	Value (%)	95% CI	Interpretation
Sensitivity	56/63	88.9	78.4-95.4	Detection of residual viable tumor
Specificity	17/21	81.0	58.1-94.6	Correct identification of pathological complete response
Positive predictive value	56/60	93.3	83.8-98.2	Probability of residual tumor when MRI was positive
Negative predictive value	17/24	70.8	48.9-87.4	Probability of no residual tumor when MRI was negative
Accuracy	73/84	86.9	77.8-93.3	Overall diagnostic agreement with histopathology

**Table 5:** Association between structured magnetic resonance imaging response category and histopathological outcome. This table shows the association between structured post-treatment magnetic resonance imaging response category and histopathological outcome in the surgically treated cohort. Data are represented as numerator/denominator and percentage. The association was assessed using the Pearson chi-square test and was statistically significant (χ² = 37.644, p < 0.001). A p-value less than 0.05 was considered statistically significant. MRI = magnetic resonance imaging

Structured MRI response category	Residual viable tumor present, n (%)	Pathological complete response, n (%)	Total, n (%)
Incomplete or indeterminate response	56/63 (88.9%)	4/21 (19.0%)	60/84 (71.4%)
Complete or near-complete response	7/63 (11.1%)	17/21 (81.0%)	24/84 (28.6%)
Total	63/84 (75.0%)	21/84 (25.0%)	84/84 (100.0%)
Pearson chi-square test	χ² = 37.644	p < 0.001	Statistically significant

Exploratory predictive, receiver operating characteristic, and sensitivity analyses

Exploratory predictive analysis was performed in the 84 surgically treated patients with histopathological reference standard. Because the number of pathological complete response events was limited and several imaging variables were overlapping components of the structured MRI response category, formal multivariable logistic regression was considered but was not used to create a definitive clinical prediction model. Persistent intermediate T2 signal showed a strong association with residual viable tumor, with an odds ratio of 20.09 and a 95% confidence interval of 5.65-71.44. Focal diffusion restriction was also strongly associated with residual viable tumor, with an odds ratio of 17.63 and a 95% confidence interval of 4.58-67.82. Residual extramural vascular invasion and threatened mesorectal fascia were seen only in patients with residual viable tumor and not in patients with pathological complete response; therefore, Haldane-Anscombe correction was applied for odds ratio estimation. The corrected odds ratio was 23.31 for residual extramural vascular invasion and 17.48 for threatened mesorectal fascia. These findings support persistent intermediate T2 signal and focal diffusion restriction as the most stable imaging predictors of residual viable tumor, while residual extramural vascular invasion and threatened mesorectal fascia represent high-risk adverse post-treatment features.

Receiver operating characteristic analysis was performed using the ordered structured MRI response category as an ordinal predictor of residual viable tumor. The ordered response category showed good discriminatory performance, with an area under the curve of 0.860. Bootstrap internal validation showed an approximate 95% confidence interval of 0.759-0.944, supporting acceptable internal stability of the ordinal response-category model. When structured MRI response was dichotomized as incomplete or indeterminate response versus complete or near-complete response, the area under the curve was 0.849.

Sensitivity analysis was performed to evaluate the effect of the indeterminate MRI response category on diagnostic performance. In the primary analysis, indeterminate response was treated as positive for residual disease, giving sensitivity of 56/63 (88.9%), specificity of 17/21 (81.0%), positive predictive value of 56/60 (93.3%), negative predictive value of 17/24 (70.8%), and accuracy of 73/84 (86.9%). When indeterminate cases were excluded, sensitivity was 49/56 (87.5%), specificity was 17/20 (85.0%), positive predictive value was 49/52 (94.2%), negative predictive value was 17/24 (70.8%), and accuracy was 66/76 (86.8%). When indeterminate response was treated as negative, sensitivity decreased to 49/63 (77.8%), specificity was 18/21 (85.7%), positive predictive value was 49/52 (94.2%), negative predictive value decreased to 18/32 (56.2%), and accuracy was 67/84 (79.8%). These findings indicate that indeterminate post-treatment MRI findings should not be considered reassuring and should prompt multidisciplinary correlation, endoscopic reassessment, short-interval MRI, biopsy when appropriate, or close surveillance.

Interobserver agreement

Interobserver agreement was substantial for the overall imaging response category, with a kappa value of 0.78. Agreement was substantial for magnetic resonance tumor regression grade, with a kappa value of 0.72, and almost perfect for diffusion restriction assessment, with a kappa value of 0.81. Agreement was almost perfect for mesorectal fascia status, with a kappa value of 0.84, substantial for extramural vascular invasion status, with a kappa value of 0.76, and substantial for nodal response assessment, with a kappa value of 0.69. These interobserver agreement values were calculated from the independent reader assessments before consensus review. Because only summary kappa values were available for final manuscript analysis and the complete reader-by-reader contingency matrices were not retained in the extracted dataset, 95% confidence intervals for individual kappa values could not be reliably recalculated. This limitation was acknowledged in the statistical interpretation, and the final consensus assessment was used only after independent agreement analysis had been completed.

Watch-and-wait subgroup

In total 16 (16.0%) patients were selected for non-operative watch-and-wait surveillance after multidisciplinary review. Of these, 8 (50.0%) patients had complete or near-complete imaging response, 6 (37.5%) patients had incomplete imaging response but were managed according to individualized multidisciplinary decisions because of clinical, surgical, or patient-related factors, and 2 (12.5%) patients had indeterminate imaging findings requiring close follow-up. Watch-and-wait selection was not based on MRI alone and included multidisciplinary assessment with clinical examination, endoscopic findings, tumor marker status where available, patient fitness, surgical feasibility, and patient preference. During the available follow-up period, no definite early local regrowth was documented on follow-up MRI in the complete or near-complete response subgroup; however, follow-up duration was limited and not uniform across all patients. Therefore, the watch-and-wait findings were considered descriptive only and were not used to validate structured MRI as an independent tool for organ-preservation decision-making. Patients with incomplete or indeterminate findings underwent closer clinical, endoscopic, and imaging surveillance.

## Discussion

Principal findings

In this retrospective cohort study of 100 patients with rectal adenocarcinoma treated with total neoadjuvant therapy, structured post-treatment MRI provided a systematic framework for assessing treatment response and identifying clinically relevant residual disease. Complete or near-complete imaging response was seen in 32 (32.0%) patients, incomplete response was observed in 58 (58.0%) patients, and indeterminate response was observed in 10 (10.0%) patients. Among surgically treated patients, structured MRI showed good diagnostic performance for detecting residual viable tumor, with sensitivity of 88.9%, specificity of 81.0%, positive predictive value of 93.3%, negative predictive value of 70.8%, and accuracy of 86.9%. Interobserver agreement was substantial for overall response category and almost perfect for assessment of mesorectal fascia status and diffusion restriction.

The present findings should be interpreted in the context of previous MRI response-assessment literature showing that post-treatment rectal MRI is clinically useful but imperfect for distinguishing complete response from residual viable tumor. DWI may improve detection of residual disease when interpreted with high-resolution T2-weighted imaging; however, false-positive and false-negative findings can occur because fibrosis, edema, mucinous change, inflammation, and small-volume residual tumor may overlap in appearance. In the present cohort, persistent intermediate T2 signal and focal diffusion restriction were the most stable imaging predictors of residual viable tumor, while residual extramural vascular invasion and threatened mesorectal fascia represented adverse post-treatment risk features. These results support structured MRI as a standardized communication tool for multidisciplinary decision-making rather than as a stand-alone determinant of complete response or organ-preservation eligibility.

These findings support the value of a structured post-treatment MRI approach in rectal cancer, particularly in the current era of total neoadjuvant therapy, where management decisions are no longer limited to routine total mesorectal excision but may include local excision or non-operative watch-and-wait surveillance in selected patients. The patient selection process is shown in Figure 1, while the key imaging parameters and response categories used in this study are summarized in Tables 1 and 2.

Role of post-treatment magnetic resonance imaging after total neoadjuvant therapy

Total neoadjuvant therapy has changed the treatment pathway of locally advanced rectal cancer by delivering systemic chemotherapy and chemoradiotherapy before surgery, thereby improving tumor regression and increasing the possibility of organ preservation in selected responders [1-5]. In this setting, MRI has expanded from a staging tool to a response-assessment tool that directly informs multidisciplinary decision-making. Baseline MRI remains essential for defining tumor height, T stage, mesorectal fascia involvement, extramural vascular invasion, nodal disease, and sphincter complex involvement [9-11]. However, post-treatment MRI must additionally determine whether the treated tumor bed represents fibrosis, residual viable tumor, or an equivocal response pattern.

In our cohort, complete or near-complete response was characterized by predominant low T2 signal fibrosis, absence of definite focal diffusion restriction, regression of extramural disease, absence of suspicious residual nodes, and absence of threatened mesorectal fascia. These findings are consistent with previous studies showing that MRI-detected tumor regression and favorable response patterns are associated with improved oncologic outcomes [12]. Representative imaging patterns of complete or near-complete response, incomplete response, and adverse residual features are illustrated in Figure 2.

Importance of T2-weighted morphology and diffusion-weighted imaging

T2-weighted morphology remains the foundation of post-treatment rectal MRI. Predominant low T2 signal fibrosis at the previous tumor site usually indicates a favorable response, while persistent intermediate T2 signal, nodular wall thickening, irregular extramural soft tissue, or persistent tumor-like morphology suggests residual viable tumor. Nevertheless, interpretation of T2-weighted imaging after treatment can be difficult because fibrosis, edema, mucin, inflammation, and desmoplastic reaction may overlap with residual tumor.

DWI provides complementary functional information. In this study, focal diffusion restriction was strongly associated with residual viable tumor. It was present in 47 of 63 patients (74.6%) with residual viable tumor but only 3 of 21 patients (14.3%) with pathological complete response. This supports the concept that DWI should not be interpreted in isolation but should be correlated with the corresponding T2-weighted abnormality and apparent diffusion coefficient map [13-16]. A focal high signal on high b-value diffusion-weighted imaging with corresponding low apparent diffusion coefficient signal at the tumor bed was considered suspicious for residual disease, whereas diffuse, linear, or ill-defined signal without a focal apparent diffusion coefficient correlate was not considered definite residual tumor.

Residual high-risk features and surgical planning

Beyond the primary tumor bed, structured assessment of mesorectal fascia, extramural vascular invasion, nodal response, sphincter complex, levator, and pelvic sidewall disease is critical. In our study, residual extramural vascular invasion, suspicious residual nodes, and threatened mesorectal fascia were found mainly in patients with residual viable tumor. Residual extramural vascular invasion was present in 22 of 63 patients (34.9%) with residual viable tumor and in 0 of 21 patients (0.0%) with pathological complete response. Similarly, threatened mesorectal fascia was present in 18 of 63 patients (28.6%) with residual viable tumor and in 0 of 21 patients (0.0%) with pathological complete response.

These findings are clinically important because the presence of persistent extramural vascular invasion, suspicious nodes, or threatened mesorectal fascia may influence the decision for total mesorectal excision, extended surgery, lateral pelvic nodal management, or intensified follow-up. Earlier MRI studies have demonstrated the importance of mesorectal fascia and extramural disease assessment in predicting surgical margin status and outcome [10-12]. In the post-treatment setting, these findings remain important, but they must be interpreted carefully to avoid overstaging fibrosis as residual tumor.

Implications for organ preservation and watch-and-wait management

Organ-preserving strategies have gained attention because a subset of patients may achieve clinical complete response after neoadjuvant therapy and avoid immediate radical surgery [4-8]. The watch-and-wait strategy requires careful integration of clinical examination, endoscopy, carcinoembryonic antigen level, and MRI. MRI alone cannot confirm complete response with absolute certainty, but it is essential for excluding adverse deep pelvic features that may not be visible on endoscopy or digital rectal examination.

In our cohort, 16 (16.0%) patients were selected for watch-and-wait surveillance after multidisciplinary assessment. Of these, 8 (50.0%) patients had complete or near-complete imaging response, 6 (37.5%) patients had incomplete imaging response but were considered unsuitable for immediate surgery or were managed according to individualized multidisciplinary decisions, and 2 (12.5%) patients had indeterminate imaging findings requiring close follow-up. During the available follow-up period, no patient in the complete or near-complete imaging response subgroup showed definite early local regrowth on follow-up MRI. However, median follow-up duration and longer-term local regrowth outcomes were not uniformly available for all watch-and-wait patients at the time of analysis. This limitation is important because the long-term safety of organ preservation depends on accurate initial selection, strict follow-up, detection of local regrowth, and timely salvage surgery when regrowth occurs [6-8,16]. These findings reinforce the role of MRI as part of a combined response assessment pathway rather than as a standalone test.

The watch-and-wait subgroup in this study should be interpreted cautiously. Only 16 (16.0%) patients were managed non-operatively, and follow-up duration was limited and not uniform. Therefore, this study cannot validate structured MRI as an independent tool for selecting patients for watch-and-wait management or for establishing long-term oncologic safety. In clinical practice, organ-preservation decisions require integrated assessment with digital rectal examination, endoscopy, tumor marker status, patient fitness, surgical feasibility, patient preference, and multidisciplinary consensus. Structured MRI may contribute to this process by documenting favorable response patterns and adverse residual features, but it should not replace clinical, endoscopic, pathological, biochemical, and multidisciplinary assessment.

Value of structured reporting

A major practical finding of this study is that structured reporting improves completeness and clarity of post-treatment rectal cancer MRI assessment. Unstructured reports may describe the tumor bed but omit critical information such as diffusion restriction, mesorectal fascia status, extramural vascular invasion response, nodal response, sphincter involvement, or pelvic sidewall disease. Such omissions can reduce the usefulness of the report during multidisciplinary discussion.

The structured template used in this study is consistent with currently available international rectal MRI reporting recommendations in that it includes key elements emphasized by ESGAR and MERCURY-related frameworks, such as tumor-bed morphology, mesorectal fascia or circumferential resection margin status, extramural spread, extramural vascular invasion, nodal assessment, and response evaluation. It also incorporates mrTRG-related assessment of fibrosis versus residual tumor signal and DWI correlation, which are commonly used in post-treatment response assessment. However, the present template differs by consolidating these components into a single management-oriented post-treatment reporting format specifically for patients treated with total neoadjuvant therapy. In addition to standard staging and restaging parameters, it explicitly links residual tumor-bed signal, diffusion restriction, mesorectal fascia status, extramural vascular invasion response, nodal response, sphincter or levator involvement, pelvic sidewall disease, and adjacent organ involvement with practical response categories relevant to total mesorectal excision, local excision, or watch-and-wait consideration.

The structured template used in this study ensured that each relevant anatomic and functional parameter was assessed consistently. This approach is particularly important in the total neoadjuvant therapy era because the radiologist’s report may influence whether the patient proceeds to total mesorectal excision, local excision, intensified surveillance, or watch-and-wait management. The structured response categories used in Table 2 provide a practical framework for translating imaging findings into clinically meaningful categories.

Comparison with previous literature

Our findings are broadly consistent with previous work on rectal MRI response assessment. The MERCURY experience showed the prognostic value of MRI-based assessment in rectal cancer and highlighted the importance of extramural depth, circumferential resection margin, and tumor regression [10-12]. Lambregts et al. demonstrated the value of DWI in identifying complete responders after chemoradiation [13]. More recent reviews, practical guides, and multireader studies emphasize that restaging MRI must combine T2-weighted morphology, DWI, nodal assessment, mesorectal fascia evaluation, and recognition of post-treatment pitfalls, while also acknowledging reader variability and imperfect accuracy in identifying complete response [14-19,21]. Recent review evidence also emphasizes that MRI evaluation of complete response after neoadjuvant therapy remains evolving and should be interpreted with clinical and endoscopic correlation [21].

The present study adds to this literature by applying a structured response assessment template specifically in patients treated with total neoadjuvant therapy and by correlating imaging response categories with surgical and histopathological outcomes. The diagnostic performance observed in our cohort suggests that structured MRI can identify residual viable tumor with clinically useful accuracy, although the negative predictive value remains imperfect. The exploratory predictive analysis further supported the association between structured MRI features and residual viable tumor. Persistent intermediate T2 signal and focal diffusion restriction showed strong odds ratio associations with residual viable tumor, and the ordered structured MRI response category demonstrated good discriminatory performance on receiver operating characteristic analysis, with an area under the curve of 0.860. However, these findings should be regarded as exploratory and internally assessed only. Because the number of pathological complete response events was limited and several imaging variables were overlapping components of the structured response category, formal multivariable logistic regression was not used to generate a definitive clinical prediction model. Larger datasets with patient-level clinical and imaging variables are required to determine independent predictors and to externally validate any prediction model. Therefore, a complete or near-complete imaging response should not be interpreted as equivalent to pathological complete response without clinical and endoscopic correlation.

Limitations

This study has several limitations. First, it was retrospective in design and was conducted at a single institution, which limits generalizability and may introduce institutional practice bias. Second, although the cohort included 100 patients, the histopathological reference standard was available only in the 84 surgically treated patients. Patients managed with watch-and-wait surveillance did not uniformly have pathological confirmation and were not included in diagnostic performance calculations unless histopathology became available. This may introduce verification bias and selection bias, because patients selected for non-operative management were more likely to have favorable clinical, endoscopic, and imaging responses. Therefore, sensitivity, specificity, positive predictive value, negative predictive value, and accuracy should be interpreted as estimates for the surgically treated subgroup rather than for the entire cohort. Third, the watch-and-wait subgroup was small, with only 16 (16.0%) patients, and follow-up duration was limited and not uniform. Long-term outcomes such as local regrowth, distant metastasis, disease-free survival, overall survival, salvage surgery rate, and organ-preservation durability could not be reliably assessed. Therefore, this study cannot validate structured MRI as an independent tool for selecting patients for watch-and-wait management or for establishing the long-term oncologic safety of organ preservation. Fourth, although reader assessment was performed independently before consensus review, the final clinicopathological correlation was based on consensus interpretation; this may have improved apparent diagnostic performance and is a potential observer-related bias. Fifth, interobserver agreement was reported using kappa values, but 95% confidence intervals for all individual agreement values could not be reliably recalculated from the extracted summary dataset. Sixth, treatment regimens, imaging intervals, follow-up duration, baseline tumor stage, tumor location, pretreatment carcinoembryonic antigen level, and other clinical factors may influence treatment response and residual viable tumor. These variables should be incorporated into larger patient-level predictive models. Seventh, microscopic residual disease may not be detectable on MRI, even when T2-weighted imaging and DWI show a favorable response. Finally, the structured reporting template was not externally validated. Larger prospective multicenter studies with standardized MRI protocols, uniform treatment pathways, complete clinical and histopathological datasets, longer follow-up, larger watch-and-wait cohorts, and independent external validation are required before routine implementation as a validated predictive framework.

Clinical implications

The findings of this study support the use of structured post-treatment rectal MRI reporting as part of multidisciplinary response assessment after total neoadjuvant therapy. Reports should document primary tumor-bed morphology, diffusion restriction, magnetic resonance tumor regression grade, mesorectal fascia status, extramural vascular invasion response, mesorectal and lateral pelvic nodal response, sphincter complex involvement, levator involvement, and pelvic sidewall disease. These imaging findings should be integrated with digital rectal examination, endoscopy, carcinoembryonic antigen level, surgical assessment, patient-related factors, and multidisciplinary discussion before deciding on total mesorectal excision, local excision, intensified surveillance, or watch-and-wait management. The present results suggest that structured MRI can improve consistency of response documentation and help identify adverse residual features; however, they do not establish MRI as an independent decision-making tool for organ preservation. Routine implementation as a validated predictive framework should be supported by prospective multicenter studies with standardized imaging protocols, patient-level clinical modeling, longer follow-up, and external validation.

## Conclusions

Structured post-treatment rectal MRI provides a practical framework for documenting treatment response after total neoadjuvant therapy and for communicating residual tumor morphology, diffusion restriction, magnetic resonance tumor regression pattern, mesorectal fascia status, extramural vascular invasion response, nodal response, and sphincter or pelvic sidewall involvement to the multidisciplinary team. In this retrospective cohort, structured MRI showed good diagnostic performance for detecting residual viable tumor in the surgically treated subgroup, and persistent intermediate T2 signal, focal diffusion restriction, residual extramural vascular invasion, and threatened mesorectal fascia were strongly associated with residual viable tumor. Exploratory receiver operating characteristic analysis demonstrated good discriminatory performance of the ordered structured MRI response category; however, these findings should be interpreted cautiously because histopathological confirmation was not available in all patients, the watch-and-wait subgroup was small, follow-up was limited, and external validation was not performed. Structured MRI may support multidisciplinary assessment of potential organ-preservation eligibility, but it should not replace integrated clinical, endoscopic, pathological, biochemical, surgical, and multidisciplinary evaluation. Larger prospective multicenter studies with standardized MRI protocols, patient-level predictive modeling, longer follow-up, and external validation are required before routine use as a validated independent predictive framework.

## Appendices

Appendix 1: Structured post-treatment rectal MRI reporting template after total neoadjuvant therapy

Patient and Treatment Details

Patient age/sex:

Clinical indication:

Histopathology: Rectal adenocarcinoma/other:

Baseline MRI date:

Post-treatment MRI date:

Neoadjuvant treatment regimen:

Date of completion of total neoadjuvant therapy:

Interval between treatment completion and post-treatment MRI:

Planned management before MRI: Total mesorectal excision/local excision/watch-and-wait consideration/other:

Primary Tumor Bed Assessment

Tumor location: Lower rectum / mid rectum / upper rectum

Distance from anal verge: ___ cm

Craniocaudal length of treated tumor bed: ___ cm

Circumferential location: anterior / posterior / right lateral / left lateral / circumferential

Comparison with baseline MRI: marked regression / partial regression / stable disease/progression

Dominant T2-weighted appearance: predominant low T2 signal fibrosis / mixed fibrosis and intermediate signal / predominant intermediate tumor-like signal / mucinous change/edema or inflammation / indeterminate

Nodular residual wall thickening: absent/present/indeterminate

Residual extramural soft tissue: absent/present/indeterminate

Diffusion-Weighted Imaging Assessment

Focal high signal on high b-value DWI: absent/present/equivocal

Corresponding low signal on ADC map: absent/present/equivocal

Overall diffusion restriction: absent/definite focal restriction/equivocal

Site of restriction: primary tumor bed/extramural component/lymph node/other:

Interpretation: no definite residual viable tumor/suspicious for residual viable tumor/indeterminate

Magnetic Resonance Tumor Regression Pattern

Dominant regression pattern: predominant fibrosis/mixed fibrosis and residual tumor signal/predominant residual tumor signal/mucinous response/indeterminate

Suggested mrTRG-equivalent impression: favorable response/intermediate response/poor response/indeterminate

Mesorectal Fascia and Circumferential Resection Margin Risk

Shortest distance of residual tumor or suspicious extramural disease from mesorectal fascia: ___ mm

Mesorectal fascia status: clear/close/threatened/indeterminate

Threatened mesorectal fascia by residual tumor within 1 mm: absent/present/indeterminate

Fibrosis alone close to mesorectal fascia: absent/present

Extramural Vascular Invasion Response

Baseline EMVI: absent/present/indeterminate

Post-treatment EMVI appearance: absent/treated fibrotic EMVI/suspicious residual EMVI/indeterminate

Features suggesting residual EMVI: expanded vessel/nodular intravascular signal/irregular intermediate T2 signal/diffusion restriction

Mesorectal Nodal Assessment

Suspicious residual mesorectal nodes: absent/present/indeterminate

Largest suspicious mesorectal node short-axis diameter: ___ mm

Nodal morphology: smooth/irregular border/heterogeneous signal/round morphology/diffusion restriction

Interval nodal response: resolved/decreased/stable/increased/indeterminate

Lateral Pelvic Nodal Assessment

Suspicious lateral pelvic nodes: absent/present/indeterminate

Side: right/left/bilateral

Nodal station: internal iliac/obturator/external iliac/common iliac/other

Largest short-axis diameter: ___ mm

Diffusion restriction: absent/present/equivocal

Sphincter Complex and Levator Assessment

Internal sphincter involvement: absent/present/indeterminate

Intersphincteric plane involvement: absent/present/indeterminate

External sphincter involvement: absent/present/indeterminate

Puborectalis/levator ani involvement: absent/present/indeterminate

Sphincter preservation on MRI grounds: favorable/caution required/not favorable/indeterminate

Pelvic Sidewall and Adjacent Organ Assessment

Pelvic sidewall involvement: absent/present/indeterminate

Adjacent organ involvement: absent/present/indeterminate

Organ involved, if present: prostate/seminal vesicle/vagina/uterus/bladder/sacrum/other

Residual disease versus fibrosis: residual tumor suspected/fibrosis favored/indeterminate

Other Pelvic or Distant Findings Within Field of View

Ascites: absent/present

Peritoneal deposits: absent/present/indeterminate

Bone marrow lesion: absent/present/indeterminate

Pelvic collection or treatment-related change: absent/present

Other clinically relevant finding:

Final Structured MRI Response Category

Overall response category: complete or near-complete response / incomplete response / indeterminate response

Criteria supporting complete or near-complete response:

Predominant low T2 signal fibrosis: yes/no

No definite focal diffusion restriction: yes/no

No suspicious residual EMVI: yes/no

No threatened mesorectal fascia by residual tumor: yes/no

No suspicious residual mesorectal or lateral pelvic nodes: yes/no

Criteria supporting incomplete response:

Persistent intermediate tumor-like T2 signal: yes/no

Focal diffusion restriction: yes/no

Residual extramural vascular invasion: yes/no

Suspicious residual lymph nodes: yes/no

Threatened mesorectal fascia: yes/no

Persistent sphincter, levator, pelvic sidewall, or adjacent organ involvement: yes/no

Criteria supporting indeterminate response:

Equivocal T2 signal: yes/no

Equivocal diffusion signal: yes/no

Mucinous change, edema, inflammation, or artifact limiting interpretation: yes/no

Suggested Impression Format

Structured post-treatment rectal MRI shows:

Overall response category: complete or near-complete response/incomplete response/indeterminate response

Primary tumor bed: predominant fibrosis/residual tumor-like signal/equivocal change

Diffusion restriction: absent/present/equivocal

Mesorectal fascia: clear/close/threatened/indeterminate

EMVI response: absent/treated fibrotic/suspicious residual/indeterminate

Nodal response: no suspicious residual nodes/suspicious mesorectal nodes/suspicious lateral pelvic nodes/indeterminate

Sphincter, levator, pelvic sidewall, or adjacent organ involvement: absent/present/indeterminate

Recommendation: correlate with digital rectal examination, endoscopy, tumor marker status, surgical findings, and multidisciplinary tumor board discussion for total mesorectal excision, local excision, or watch-and-wait consideration.
